# Some Dietary Phenolic Compounds Can Activate Thyroid Peroxidase and Inhibit Lipoxygenase-Preliminary Study in the Model Systems

**DOI:** 10.3390/ijms22105108

**Published:** 2021-05-12

**Authors:** Ewa Habza-Kowalska, Agnieszka A. Kaczor, Damian Bartuzi, Jacek Piłat, Urszula Gawlik-Dziki

**Affiliations:** 1 Department of Biochemistry and Food Chemistry, University of Life Sciences, Skromna Str. 8, 20-704 Lublin, Poland; ewa.habza1@gmail.com; 2 Department of Synthesis and Chemical Technology of Pharmaceutical Substances with Computer Modeling Laboratory, Faculty of Pharmacy, Medical University of Lublin, 4A Chodzki St., 20-093 Lublin, Poland; agnieszka.kaczor@umlub.pl (A.A.K.); damian.bartuzi@umlub.pl (D.B.); 3 School of Pharmacy, University of Eastern Finland, Yliopistonranta 1, P.O. Box 1627, FI-70211 Kuopio, Finland; 4 Department of General Surgery, Transplantology and Clinical Nutrition, Medical University of Lublin, Jaczewskiego Str. 8, 20-090 Lublin, Poland; jacek.pilat@umlub.pl

**Keywords:** thyroid peroxidase (TPO), lipoxygenase (LOX), inhibition, dietary polyphenols, antioxidant activity, interactions, isobolographic analysis

## Abstract

The presented research concerns the triple activity of *trans*-cinnamic (tCA), ferulic (FA) and syringic acids (SA). They act as thyroid peroxidase (TPO) activators, lipoxygenase (LOX) inhibitors and show antiradical activity. All compounds showed a dose-dependent TPO activatory effect, thus the AC_50_ value (the concentration resulting in 50% activation) was determined. The tested compounds can be ranked as follows: tCA > FA > SA with AC_50_ = 0.10, 0.39, 0.69 mM, respectively. Strong synergism was found between FA and SA. The activatory effects of all tested compounds may result from interaction with the TPO allosteric site. It was proposed that conformational change resulting from activator binding to TPO allosteric pocket results from the flexibility of a nearby loop formed by residues Val352-Tyr363. All compounds act as uncompetitive LOX inhibitors. The most effective were tCA and SA, whereas the weakest was FA (IC_50_ = 0.009 mM and IC_50_ 0.027 mM, respectively). In all cases, an interaction between the inhibitors carboxylic groups and side-chain atoms of Arg102 and Arg139 in an allosteric pocket of LOX was suggested. FA/tCA and FA/SA acted synergistically, whereas tCA/SA demonstrated antagonism. The highest antiradical activity was found in the case of SA (IC_50_ = 0.22 mM). FA/tCA and tCA/SA acted synergistically, whereas antagonism was found for the SA/FA mixture.

## 1. Introduction

Hashimoto’s thyroiditis (HT) (i.e., chronic lymphocytic thyroiditis) is an autoimmune disease, in the course of which the thyroid gland is attacked and destroyed by the immune system. The resulting inflammation often leads to an underactive thyroid gland (i.e., hypothyroidism). The disease affects between 0.1% and 5% of the adult population in Western countries [1]. Unfortunately, the major antigen in human Hashimoto’s disease is thyroid peroxidase (thyroperoxidase, TPO, EC 1.11.1.1–14), an enzyme that participates in the synthesis of thyroid hormones. Anti-TPO antibodies induce complement-dependent cytotoxicity. Furthermore, antibodies against complement (anti-C1q) are detected in patients with Hashimoto’s disease. They are correlated with thyroid-stimulating hormone (TSH) levels. Thus, many patients with congenital hypothyroidism have problems related to the synthesis or iodination of thyroglobulin (TG), which is connected to TPO deficiency [2]. Disturbances in the functioning of the thyroid have many effects on human health, such as inflammation and oxidative stress [3]. Our previous studies showed that the selected pure polyphenolic substances can affect TPO activity [4].

Inflammation is a natural defense mechanism against pathogens, and it is associated with many disorders, such as microbial and viral infections and exposure to allergens, radiation and toxic chemicals, as well as autoimmune and chronic diseases. There are various possible mechanisms for the anti-inflammatory effects of bioactive compounds, including the inhibition of lipoxygenases (LOX) that catalyze the oxygenation of polyunsaturated fatty acids into potent signal molecules involved in inflammatory processes [5]. LOXs participate in eicosanoid syntheses, such as prostaglandins or nonclassic eicosanoids. The LOX pathway of arachidonic acid metabolism generates reactive oxygen species (ROS), which, together with other arachidonic acid metabolites, play a role in inflammation and tumor growth [1]. LOX activity is related to oxidative stress (OS) in the human body. OS refers to a condition in which the balance between the antioxidant protective system and the production of ROS is disturbed. The oxidative protection system can be effectively supported by various types of exogenous compounds with antioxidant activity. Some of the most effective antioxidants are phenolic compounds, the group of secondary plant metabolites with documented antioxidant activity [6]. The antioxidant effects of polyphenols are mainly due to their redox potential, which enables them to act as reducing agents, donors of hydrogen and quenchers of single oxygen. They also may be inhibitors of free-radical reaction through the inhibition of lipid radical formation and disruption of the propagation of chain auto-oxidation reactions [2].

Our previous studies showed that polyphenols can affect TPO activity [4]. During in vitro and in silico screening tests, some phenolic acids commonly found in food have been found to activate TPO. Ferulic acid can be found, especially in cereals, fruits and vegetables. For human health, it effectively scavenges free radicals and inhibits lipid peroxidation [7]. Some studies mention its cardioprotective effect and its inhibition of tumor promotion [8]. Sources of cinnamic acid are vegetable oils, berries and citrus juices. This compound shows mainly gastroprotective effects [9]. Syringic acid shows hepatoprotective, antihyperglycemic and antimicrobial activity and can be found in several types of alcohols: brandy, rum, whisky, nut liquors and fortified wines. Good sources of these polyphenols also include cereals, dried fruits and vegetable oils [8].

Since phenolic compounds are widely known for their anti-inflammatory and antioxidant properties, it has been hypothesized that TPO activators can inhibit LOX activity and have antioxidant effects.

A factor that essentially influences the final effect of biologically active compounds, especially from food systems, is their interaction. Therefore, another hypothesis has been made, that the interactions of the test compounds may substantially modulate their action as TPO and LOX effectors. To investigate possible interactions, the isobolographic method was used. Isobolographic analysis is a scientific approach that graphically represents antioxidant interactions, thereby facilitating their visual evaluation. It makes the interactions of sample combinations succinct and clear and has been widely established as a gold standard for testing pharmacological interactions with various combinations of fixed fractions [10].

Thus, this research aimed to estimate TPO-activatory and LOX-inhibitory effects, as well as the antiradical potential of ferulic, syringic and *trans*-cinnamic acids. To elucidate the mechanism of the effectors’ action, in silico studies were carried out. Another part of this paper consists of the analysis of the kind and strength of possible interactions between tested compounds.

## 2. Results and Discussion

### 2.1. TPO Assay

**Figure 1:**
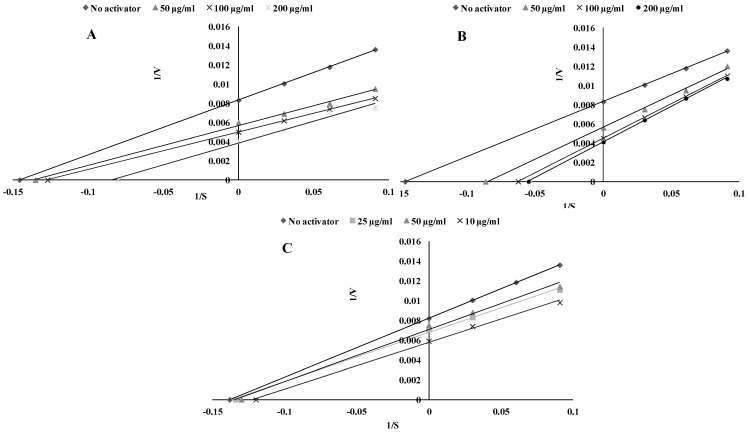
Activatory effect of ferulic acid (**A**), syringic acid (**B**) and *trans*-cinnamic acid (**C**) on thyroid peroxidase TPO activity. Plots are expressed 1/velocity versus 1/guaiacol [μg/mL] without or with activators in a reaction solution.

A previous study [4] contained the first part of the investigation, which showed the TPO-inhibitory potential of pure polyphenols. During the next stage of investigation, activatory effects were observed for some other polyphenols. This paper contains the second part, which contains the TPO activatory effect of the following phenolic compounds: ferulic (FA), syringic (SA) and *trans*-cinnamic acids (tCA). To present this effect, Lineweaver–Burk plots were prepared. A plot without the polyphenol addition, which is above the plots with addition of polyphenols, shows activation (Figure 1).

Tested compounds show a dose-dependent activatory effect, which allowed the determination of the AC_50_ value—the concentration of effector at which 50% activation was obtained. Taking into account this parameter, the tested phenolic acids can be ranked as follows: tCA > FA > SA. The kinetic parameters of TPO activation were presented in Table 1.

The data showing the influence of phenolic compounds on TPO activity are still incomplete. Most of the studies available concern the inhibitory effect of phenolic compounds on the activity of TPO. TPO-inhibitory potential was described for flavones apigenin, chrysin, vitexin and baicalein, present in parsley, cherries, thyme, olives, tea and broccoli [11]. The flavanols kaempferol, quercetin, fisetin, morin, myricetin and rutin, present in a wide range of food sources such as kale, onions, tomatoes, cherries, apples and red wine, together with the flavanones naringin and naringenin, can also inhibit tyrosine iodination by TPO with varying potencies [12,13]. The antithyroid potential of dihydroxybenzoic acid is also described [14]. When TPO activity is inhibited, reducing thyroid hormone synthesis, a compensatory increase in TSH may be observed; this may lead to goiter, especially when these compounds are consumed in high quantities [11]. To the best of our knowledge, there are no data on phenolic TPO activators in the available literature.

### 2.2. Molecular Modeling of TPO Activation

To study TPO activation, a new model of the protein was elaborated. The new model was significantly improved compared to the previous version [4].

The activatory activity of the studied compounds on TPO may be nonspecific and result from their antioxidant properties. In this manner, they may neutralize reactive oxygen species and protect the enzyme against damage. On the other hand, the activatory effect can be specific and result from small-molecule interaction with an allosteric or regulatory site of the enzyme, leading to protein conformational change and enhancing its catalytic activity. Here, such binding sites were identified using PARS and Fpocket online tools and molecular docking with Glide was used to discriminate between the pockets.

**Figure 2:**
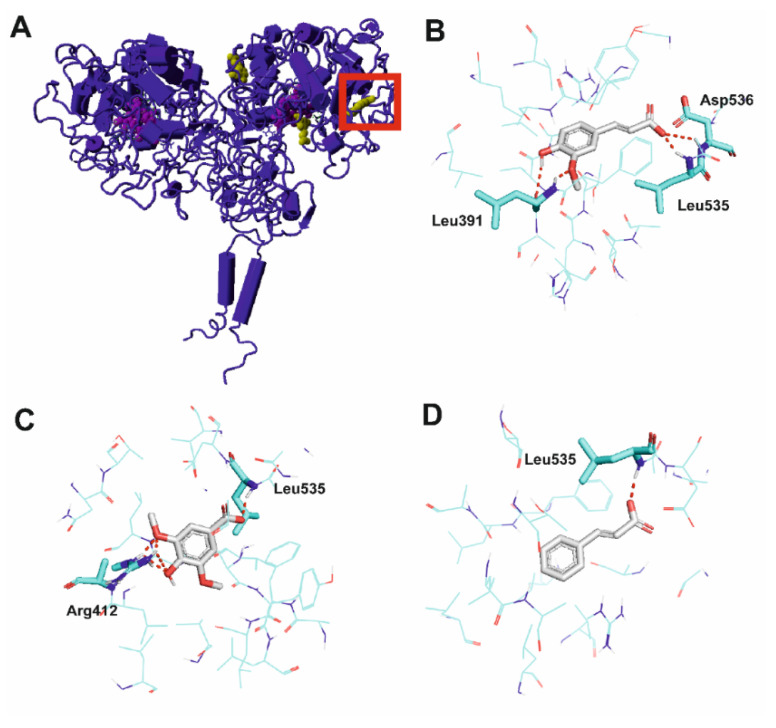
Molecular interactions of activators with thyroid peroxidase (TPO). (**A**) Ferulic acid bound to three considered binding pockets. The protein is shown in dark blue cartoon representation, ferulic acid in yellow ball representation and heme in magenta ball representation. The selected pocket is marked with a red square. (**B**) Ferulic acid, (**C**) syringic acid and (**D**) *trans*-cinnamic acid in a binding pocket of TPO. The protein is shown with cyan carbon atoms in wire representation with the most important residues shown as sticks. Activators depicted with gray carbon atoms in stick representation. Polar bonds are shown as red dashes. Nonpolar hydrogen atoms omitted for clarity.

Three potential binding cavities were considered most likely to act as regulatory sites for TPO activation: (i) in the vicinity of the heme-binding site, centered around Pro245, Arg370 and Phe523; (ii) on the enzyme dimer interface, centered around Arg175 and Trp176 from one subunit and Trp176, Asp474, Asn478, Lys662 and Asp66 from the other subunit; and (iii) at the binding site centered around Arg412 and Asp536, as shown in Figure 2A. Molecular docking indicated that the highest Glide scores were obtained for all compounds at the binding site involving Arg412 and Asp536. Moreover, docking scores to this site only corresponded to the order of Vmax values. Thus, this pocket, marked in a red square in Figure 2A, was selected for further analysis. Figure 2B–D shows the interactions of the studied compounds with the chosen binding cavity. In the case of all activators, there is an interaction between their carboxylic groups and the main chain atoms of Leu535 and their orientation in the binding pocket is similar. Ferulic acid displays additional interactions between its carboxylic group and the main chain atoms of Asp536 and between its methoxy and hydroxy groups and the main chain atoms of Leu391 (Figure 2B). Syringic acid has its methoxy and hydroxy moieties involved in hydrogen-bond interactions with the side-chain atoms of Arg412 (Figure 2C), while *trans*-cinnamic acid does not form any additional contacts (Figure 2D).

It can be hypothesized that a conformational change resulting from activators binding to this TPO binding pocket results from the flexibility of a nearby loop formed by residues Val352-Tyr363.

### 2.3. LOX Assay

As inflammation occurs in HT, the next step was to determine the effect of TPO activators on the activity of LOX, one of the main enzymes associated with the occurrence of inflammation and oxidative stress. The similarity in inhibition behavior between soybean LOX-1 and human 5-LOX has been observed, and soybean LOX (sLOX) type 1b has been used for the evaluation of LOX inhibition in drug screening for years [15].

**Figure 3:**
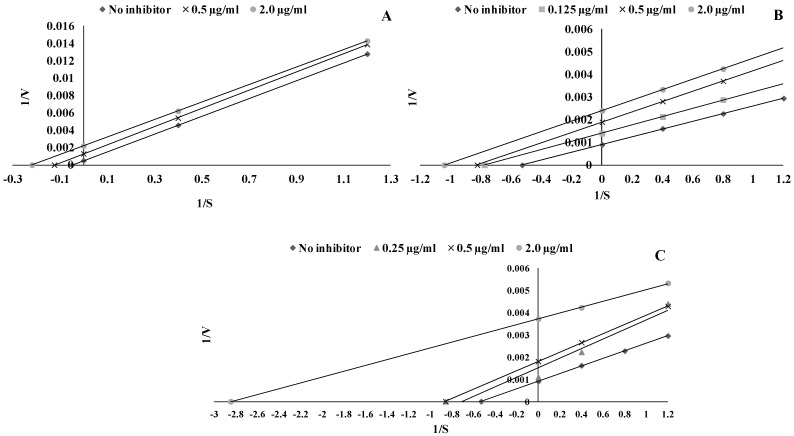
Lineweaver—Burk double reciprocal plots for the inhibition of lipoxygenase (LOX) by ferulic acid (**A**), syringic acid (**B**) and *trans*-cinnamic acid (**C**). Plots are expressed 1/velocity versus 1/linoleic acid [μg/mL] without or with inhibitors in a reaction solution.

To illustrate this influence, Lineweaver–Burk plots were prepared. As presented in Figure 3, all of the used phenolic acids showed an inhibitory effect. All tested compounds act as uncompetitive LOX inhibitors (Figure 3). The kinetic parameters of LOX inhibition were presented in Table 2. The most effective LOX inhibitors were *trans*-cinnamic and syringic acids with IC_50_ = 0.009 mM, whereas ferulic acid was the weakest LOX inhibitor (IC_50_ = 0.027 mM).

In our previous studies [7], it was found that ferulic acid acts as a competitive inhibitor of LOX. The difference may be due to the type of enzyme used for the test and the concentration of the reacting components. The obtained inhibitory potential of pure ferulic acid and *trans*-cinnamic acid are in accordance with Devi et al. [16], where these polyphenols showed 74.4 and 66.6% of inhibition respectively but in different concentration than in the presented study. The weak activity of ferulic acid as a LOX inhibitor is confirmed by the studies by Landberg et al. [5], which showed that low or no inhibition was observed with avenanthramides containing ferulic or *para*-coumaric acid. Another study provided by Saleem et al. [17] showed the LOX inhibitory activity (34.8% of inhibition) of extracts from *Filago germanica*, wherein syringic acid was detected in the amount of 2.23 µg/mL.

### 2.4. Molecular Modeling of LOX Inhibition

As shown in the experimental part, all tested phenolic acids display an uncompetitive type of inhibition. Uncompetitive inhibitors bind to the enzyme–substrate complex only. The binding of the substrate can result in a conformational change allowing the inhibitor to bind, or the inhibitor binds to the enzyme-bound substrate directly. In all cases, the inhibitor binds in an allosteric pocket and does not compete with the substrate for a binding site.

**Figure 4:**
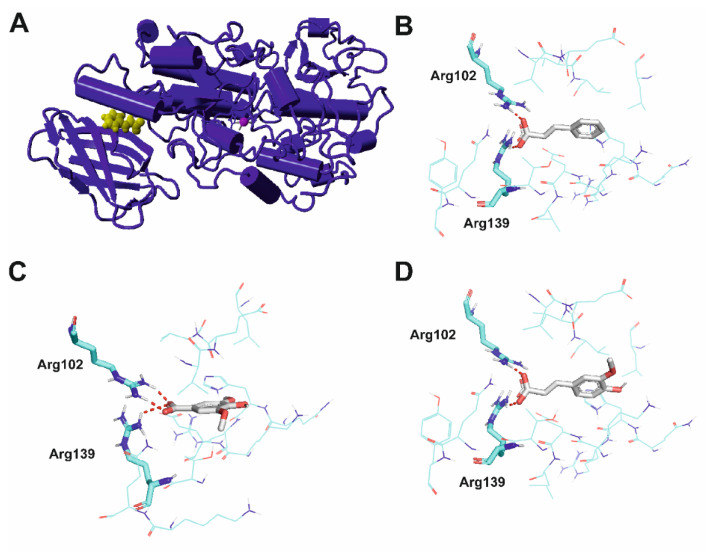
Molecular interactions of inhibitors with 5-lipoxygenase (5-LOX). (**A**) *trans*-cinnamic acid bound to an allosteric binding pocket (general view). The protein is shown in dark blue cartoon representation, *trans*-cinnamic acid in yellow ball representation and iron ion in magenta ball representation. (**B**) *trans*-cinnamic acid, (**C**) syringic acid and (**D**) ferulic acid in a binding pocket of 5-LOX. The protein is shown with cyan carbon atoms in wire representation with the most important residues shown as sticks. Inhibitors are depicted with gray carbon atoms in stick representation. Polar bonds are shown as red dashes. Nonpolar hydrogen atoms omitted for clarity.

Similarly as in the case of TPO, searching for potential binding pockets was performed using PARS and Fpocket. A number of potential binding sites were identified, including one situated between the membrane-binding and catalytic domains of 5-LOX also found in an X-ray structure of 5-LOX in complex with an allosteric modulator AKBA, a pentacyclic triterpene acid (PDB ID: 6NCF [18]. According to the PARS online tool, this pocket affects protein flexibility. This binding pocket is shown in Figure 4A and was used for further analysis.

Figure 4B–D shows the details of the interactions of the studied inhibitors with the allosteric pocket of 5-LOX. In all compounds, there is an interaction between their carboxylic groups and the side-chain atoms of Arg102 and Arg139. The mechanism of inhibition may be similar to that reported for AKBA, in which AKBA is wedged in a deep groove between the amino-terminal and catalytic domains and induces a conformational change of the protein which results in enzyme inhibition [18].

### 2.5. Antiradical Analysis

Under normal physiological conditions, oxidative homeostasis is autoregulated by the thyroid gland. Excessive ROS level can disrupt this balance, which can influence thyroid enzyme activity. However, this mechanism is still unclear [19]. Therefore, it is justified to determine the antioxidant potential of the tested compounds.

For the antioxidant activity of tested phenolic compounds evaluation, the ABTS radical scavenging assay was used. ABTS may be used to determine the activity of both hydrophilic and hydrophobic antioxidants; it is not affected by ionic strength, and it reacts with most antiradical compounds [20]. The lowest radical scavenging ability was shown by *trans*-cinnamic acid (2.12 mM). The highest value of this parameter showed syringic acid (0.22 mM) (Table 3).

As expected, tested compounds showed high radical scavenging potential. The obtained results are significantly higher than those obtained by Samsonowicz et al. [21], who showed the antioxidant properties of coffee substitutes containing polyphenols investigated in our study (*trans*-cinnamic acid, ferulic acid and syringic acid). The paper showed that the ABTS radical scavenging activity values were in the range of 0.021–0.066 mg dw/mL. On this basis, we can conclude that pure polyphenolic substances show higher ABTS radical scavenging potential than substances contained in plant food sources.

### 2.6. Interaction Assay

The next step of the investigation was to estimate the type of mixed-polyphenol interactions and their influence on TPO and LOX enzymatic activity.

**Figure 5:**
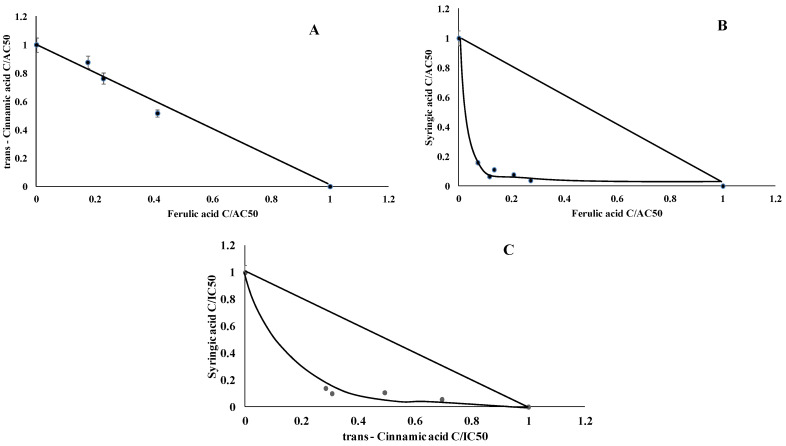
Dose-normalized isobolograms for chosen phenolic acids with thyroid peroxidase activation activity. (**A**) *trans*-cinnamic acid and ferulic acid, (**B**) syryngic acid and ferulic acid, (**C**) syringic acid and *trans*-cinnamic acid).

Isobolographic analysis of the TPO activators showed that tCA and FA (Figure 5A) acted additively; SA and FA (Figure 5B) showed strong synergism, whereas SA and tCA (Figure 5C) acted synergistically. All types of interactions were expressed as a CI value [10], which explains the strength of the interactions. As the CI values reveal, pure chemicals exhibited synergism and strong synergism for TPO (Table 4).

**Figure 6:**
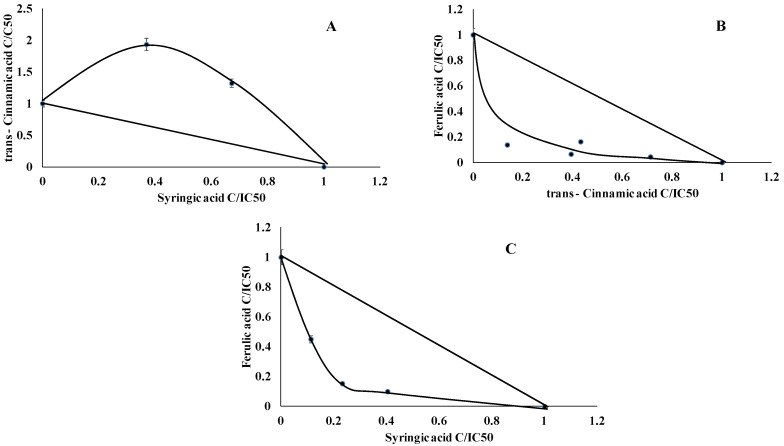
Dose-normalized isobolograms for chosen phenolic acids with lipoxygenase inhibitory activity. (**A**) *trans*-cinnamic acid and syryngic acid, (**B**) ferulic acid and *trans*-cinnamic acid, (**C**) ferulic acid and syryngic acid).

Isobolographic analysis of the LOX enzyme showed that *trans*-cinnamic acid and syringic acid acted antagonistically (Figure 6A), and FA/tCA and FA/SA acted synergistically, (Figure 6B,C). The CI values are presented in Table 5.

There are many scientific reports concerning interactions between drug components. At present, the clinically combined use of drugs is extremely common because the therapeutic effect of combined drugs is often better than that of a single drug [22]. The same effect was observed for the phytochemicals in fruits and vegetables that are responsible for their potent antioxidant and anticancer activities, and the benefit of a diet rich in fruits and vegetables is attributed to the complex mixture of phytochemicals present in whole foods [12]. One effective method for interactions assay is isobolographic analysis. It is useful to determine the interactions between mixtures consisting of two or three components [22]. It has also been shown to be useful in studying the interactions of bioactive plants and food ingredients [23].

Our previous studies used this method on pure polyphenolic compounds and plant extracts against TPO and LOX [4,24]. Studies [7] showed the synergistic action of ferulic and chlorogenic acids as LOX inhibitors. The same kind of interaction was found between chlorogenic and cinnamic acid [25], as well as chlorogenic and vanillic acid [26]. So far there is no information on TPO activators and their interactions in the literature.

**Figure 7:**
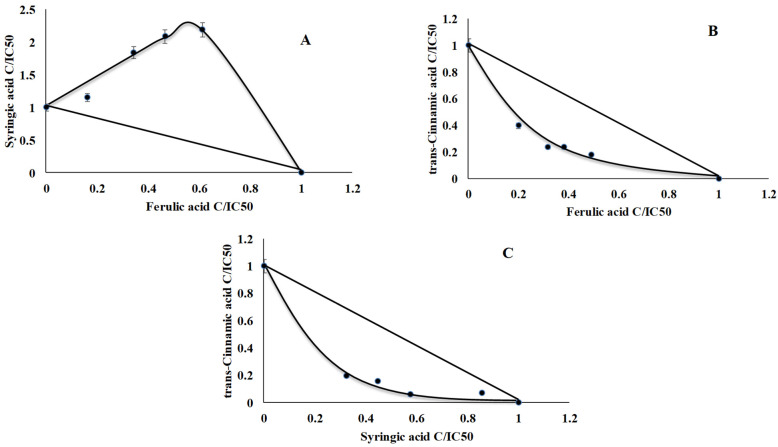
Dose-normalized isobolograms for chosen polyphenols with antiradical activity. (**A**) syryngic acid and ferulic acid, (**B**) *trans*-cinnamic acid and ferulic acid, (**C**) *trans*-cinnamic acid and syryngic acid).

The antiradical potential of phenolic compounds is well known [27], however, investigations concerning the interactions between them are still quite rare. In our study synergism was found in FA/ tCA and tCA/SA mixtures (CI = 0.63 and 0.69, respectively), while FA and SA acted antagonistically (CI = 2.21) (Figure 7, Table 6).

During research on antiradical activity, antagonism was found between chlorogenic acid and cinnamic acid [25], chlorogenic acid and vanillic acid [26], and chlorogenic acid and caffeic acid [28]. On the other hand, chlorogenic acid and ferulic acid acted synergistically as hydroxyl radical scavengers [29].

As shown in the present investigation, all tested phenolic acids show triple activity. They effectively activate TPO and inhibit LOX activity, demonstrating at the same time antioxidant potential. To the best of our knowledge, there are no reports of phenolic TPO activators with correlated anti-inflammatory and antioxidant potential in the literature to date. Most importantly, all TPO activators mentioned in the paper belong to the group of dietary polyphenols, commonly found in food of plant origin. However, the key issue here is bioavailability. Bioavailability measures the degree of an active compound/drug that reaches blood circulation and is therefore available at the site of action. As a natural antioxidant, tCA plays an important role in reducing the risk of chronic diseases, delaying ageing and improving immunity. However, the application of tCA is reduced by its poor water solubility and low oral bioavailability [30]. Previous reports indicate tCA is absorbed by all the gastrointestinal organs of the rat digestive tract and recovered in urine; however, the lowest rate of absorption was in the stomach [31]. Low plasma concentrations are most likely due to limited absorption, intensive metabolism and/or fast elimination of tCA and its derivatives from circulation. These effects may not be sufficient to produce significant in vivo biological effects. To increase the bioavailability of tCA, new formulations have been prepared in which tCA is entrapped into solid and liquid particles [30,32]. High bioavailability of FA (about 50%) was found when FA was perfused as a free pure compound in the rat intestine. The urinary excretion of FA in humans was also high (19–98%) when FA is present in its free form, for example from beer [33]. Although the main source of ferulic acid is cereals, the limited bioavailability of FA from the cereal matrix is due to the embedding of FA in the indigestible polysaccharides of the cell walls [34]. Recent pharmacological studies demonstrated that SA possesses various activities, including antitumor, chemoprevention against skin cancer and antithrombotic activity [35]. Nevertheless, the lipophilicity of SA coupled with its rapid excretion in vivo results in low bioavailability and poor therapeutic effect. On the other hand, the absolute bioavailability of SA in blood samples of rabbits was found to be 86.27% [35].

Food-derived phytochemicals with multidirectional biological properties have already been tested for potential use in a wide range of diseases, including diabetes; viral, microbial and parasitic infections; inflammation; cardiac and psychiatric disorders, as well as cancer [11]. However, little is known about the impact of phytochemicals on thyroid function.

## 3. Materials and Methods

### 3.1. Chemicals

Sucrose (α-D-glucopyranosyl-(1–4)-β-D-fructofuranoside), tris (1,3-propanediol-2-amino-2-hydroxymethyl), KCl, NaCl, MgCl_2_, 90% ethanol, NaOH, guaiacol (2-methoxyphenol), H_2_O_2_ (hydrogen peroxide), ABTS (2,2′-azinobis-(3-ethylbenzothiazoline-6-sulfonic acid), soybean lipoxygenase type 1-B (LOX), *trans*-cinnamic acid, syringic acid and ferulic acid were purchased from Sigma-Aldrich Company (Poznan, Poland). All other chemicals were of analytical grade.

### 3.2. Preparation of Phenolic Acids Solutions

Syringic acid, *trans*-cinnamic acid and ferulic acid were diluted in ethanol to the concentrations 0.125 µg/mL, 0.25 µg/mL, 0.5 µg/mL, 1.0 µg/mL, 2.0 µg/mL, 25 µg/mL, 50 µg/mL, 100 µg/mL and 200 µg/mL and were used for further assay.

### 3.3. Molecular Modeling

#### 3.3.1. Small Molecules Modeling

The studied compounds were downloaded from PubChem (syringic acid [36], trans-ferulic acid [37] and *trans*-cinnamic acid [38]) and modelled using LigPrep [39] from the Schrödinger suite of software (Release 2020–4). To determine protonation states at the physiological pH, the Epik [40] module of the Schrödinger suite of software was applied.

#### 3.3.2. Protein Models

Due to the lack of plausible templates for the complete TPO structure, the construction of a homology model of TPO including its transmembrane domain had to be performed in several steps, which included using preliminary models for positioning templates in space.

In the first step, preliminary homology models of the catalytic, MPO-like domain and the transmembrane domain were prepared with Modeller 9.23 [41]. Human promyeloperoxidase (proMPO, PDB ID: 5MFA) was used as a template for the dimer of the MPO-like domain, due to the high sequence similarity and high resolution of the X-ray structure (1.2 Å). For the transmembrane region, no obvious template was available. Therefore, a search for distant homologs was performed with the MPI Bioinformatics toolkit (https://toolkit.tuebingen.mpg.de accessed on 21 April 2021) [42]. After several HHpred runs, three structures were identified as possible templates (PDB IDs: 6HUM, 6ITH, 6NBY). All these structures allowed the modeling of the monomer of the transmembrane domain. To align these models in a dimer, a 2L2T PDB structure was used, based on literature data [43].

In the next step, preliminary models of the complete monomer were created with trRosetta [44]. These preparatory models were intended to serve as a scaffold for connecting the MPO-like domain and transmembrane domain. Candidate trRosetta models were superimposed with the model of the catalytic domain dimer, and the trRosetta model that allowed undisturbed dimerization was selected. Two copies of the selected trRosetta model were superimposed to two monomers of the MPO-like domain dimer, and the resulting structure was used as a spatial scaffold for further modelling.

In the final step, the trRosetta model of the whole TPO dimer was used to orient all the necessary templates in space. A homology model of TPO with its transmembrane domain was created with Modeller, using the 5MFA structure as a template for the MPO-like domain, 6HUM, 6ITH, 6NBY and 2L2T structures for the transmembrane domain, and the trRosetta model as a template for the region connecting the MPO-like domain and the transmembrane domain. Four hundred models were generated. The best model was chosen based on the DOPE score and its orientation relative to the membrane—the model with the highest DOPE score, not hindering the membrane plane obtained from the Orientations of Proteins in Membranes (OPM) database record for a 2L2T structure [45] was chosen for further studies.

After the homology modelling stage, the obtained model was refined with a 100 ns molecular-dynamics simulation using Gromacs 2019 [46]. The model was immersed in a membrane composed of POPC, POPE and cholesterol, using the orientation of a 2L2T structure from the OPM database as a template. The simulation box was prepared with CHARMM-GUI [47]. A CHARMM36m force field was used. An NPT ensemble with a timestep of 2 fs was used for the production run.

Regarding 5-lipoxygenase (5-LOX), an X-ray structure of the human enzyme in complex with allosteric modulator AKBA, a pentacyclic triterpene acid (PDB ID: 6NCF [18], was used after applying the necessary mutations.

The structures of the biomolecules were preprocessed using the Protein Preparation Wizard of Maestro Release of the Schrödinger software [48] to optimize the hydrogen bonding network and to remove any possible artifacts as reported previously [49].

#### 3.3.3. Binding Site Identification and Molecular Docking

Two online tools, i.e., PARS [50] and Fpocket [51], were used for putative binding site detection. PARS relies on normal mode analysis and is a simple and fast tool, which queries protein dynamics and structural conservation to find pockets on a protein structure that may exert a regulatory effect upon the binding of a small-molecule ligand. Fpocket is a binding site detection package based on Voronoi tessellation and alpha spheres [52] built on top of the publicly available package Qhull. Binding pockets identified simultaneously by both tools were used for molecular docking. Molecular docking was performed using the standard precision (SP) approach of Glide [53] from the Schrödinger suite of software. Grid files were generated at default settings. Fifty poses were generated for each binding site and each ligand. The final poses were selected by visual inspection. Discrimination between binding sites was carried out based on scoring values. PyMol v. 2.3.3 [53] and Yasara Structure [54] were used for visualization of the results.

### 3.4. Enzymatic Assay

#### 3.4.1. TPO Preparation

The assay was prepared according to Jomaa et al. [55], with some modifications. Porcine thyroid glands were purchased at a slaughterhouse (Lublin, Poland) and stored at −20 °C until used. The frozen thyroid gland was minced with a fork. The mince was suspended in a buffer containing 0.25 M sucrose, 2 mM tris–HCl, 100 mM KCl, 40 mM NaCl and 10 mM MgCl_2_ (pH 7.4) and homogenized using Philips homogenizer. The thyroid gland was centrifuged two times at 4000 RPM per 15 min at a temperature + 4 °C. The enzyme protein was then salted out to 60%. The supernatant was stored at − 20 °C until used.

#### 3.4.2. TPO Assay

The assay was used according to Jomaa et al. [55], with some modification. The measurement was made using a plate spectrophotometer (BioTek, Model Epoch2TC, Winooski, VT, USA) in 96–well plates at a wavelength of 470 nm. Absorbance readings were recorded every minute for a total of 3 min at 37 °C. TPO activity is expressed as a change of absorption per minute. All measurements were performed in three replicates. The AC_50_ (activator concentration) values were calculated at fitted models as the concentration of the tested compound providing 50% of the activation based on a dose-dependent mode of action.

#### 3.4.3. Inhibition of Lipoxygenase Activity (LOXI)

The inhibition of LOX with linoleic acid as a substrate was measured spectrophotometrically, based on Axelrod et al. [56] adopted for microplate reader (Epoch 2 Microplate Spectrophotometer, BioTek Instruments, Winooski, VT, USA). Measurement was made at a wavelength of 234 nm. One unit of LOX activity was defined as an increase in absorbance of 0.001 per minute at 234 nm. All measurements were performed in four replicates.

The IC_50_ (inhibitor concentration) values were calculated at fitted models as the concentration providing 50% of activity was based on a dose-dependent mode of action.

### 3.5. In Vitro Antiradical Capacity Assay

ABTS radical scavenging activity was determined according to Re et al. [57] with slight modifications using a microplate spectrophotometer (BioTek, Model Epoch2TC, Winooski, VT, USA) after 15 min of incubation at room temperature.

The IC_50_ (inhibitor concentration) values were calculated at fitted models as the concentration providing 50% of activity was based on a dose-dependent mode of action.

### 3.6. Isobolographic Analysis

Dose-normalized isobolograms were performed according to Chou [10]. Tested solutions were mixed in various volume ratios: 1:4, 4:1, 3:2, 2:3, 1:1. Results (type and strength of interactions) were showed as normalized isobolograms and described by combination index (CI). The quantification of interaction was done by the general Equation (1) for n-drug combination at x% inhibition using the CI for interaction interpretation:

(1) $$\mathrm{CI}=\frac{{\left(D\right)}_{1}}{{\left(D_{x}\right)}_{1}}+\frac{{\left(D\right)}_{2}}{{\left(D_{x}\right)}_{2}}={\frac{1}{\left(\mathrm{DRI}\right)}}_{1}+\frac{1}{{\left(\mathrm{DRI}\right)}_{2}}$$

where: CI is the sum of the dose of drugs that exerts x% inhibition in a combination. In the denominator, (Dx) is for D “alone” that inhibits a system x%. When CI is lower than 1, it indicates synergy; when CI is equal to 1, it indicates addition; when CI is higher than 1, it indicates antagonism.

### 3.7. Statistical Analysis

All experimental results were the mean ± SD of four parallel measurements, and data were evaluated by a two-way analysis of variance (Tukey test) using Statistica 6.0 software (StatSoft, Inc., Tulsa, OK, USA). The statistical tests were carried out at a significance level of α = 0.05. 

## 4. Conclusions

The use of phytochemicals can be a widely accepted and inexpensive way to support the treatment and prevention of many diseases, including thyroid disorders. Importantly, this solution is available to both poor societies that do not have access to expensive drugs and developed societies that struggle with the problem of ageing societies and rising health care costs. Our in silico results indicate that both activatory and inhibitory effects on TPO and LOX, respectively, are mediated by compounds’ interaction with the allosteric site of the enzymes. Further studies will be performed to address the mechanism of synergistic, additive or antagonistic interactions between the studied dietary phenolic compounds.

However, it should be kept in mind that the results obtained during in vitro tests in model systems may, to a varying degree, translate into actual effects obtained during in vivo tests, therefore, the results presented in this paper are treated as preliminary and will be verified during further extensive research.

## Figures and Tables

**Table 1:** K_m_, V_max_ and AC_50_ values of the chosen phenolic acids on thyroid peroxidase (TPO) activity, *n* = 9.

Compound	K_m_ [mM]	V_max_ [ΔAU/min]	AC_50_ [mM]
Guaiacol ^1^	0.06 ± 0.003	120.5 ± 5.38 ^d^	-
*trans*–cinnamic acid	-	128.2 ± 4.12 ^c^	0.10 ± 0.005 ^c^
Syringic acid	-	243.9 ± 9.54 ^b^	0.69 ± 0.034 ^a^
Ferulic acid	-	294.1 ± 9.12 ^a^	0.39 ± 0.019 ^b^

^1^ TPO substrate. Values are expressed as the mean ± SD; means with different letter superscripts (a–d) in the columns are significantly different (α = 0.05).

**Table 2:** Mode of inhibition, K_i_ (K_m_ without inhibitor), V_max_ and IC_50_ values of chosen phenolic acids on lipoxygenase (LOX) activity.

Compound	Mode of Inhibition	K_i_ [mM] (K_m_ without Inhibitor)	V_max_ [ΔAU/min]	IC_50_ [mM]
Linoleic acid *	-	0.007 ± 0.0003 ^a^	1111 ± 45.1 ^a^	-
*trans*–cinnamic acid	uncompetitive	0.009 ± 0.0004 ^c^	556 ± 10.13 ^b^	0.009 ± 0.0004 ^b^
Syringic acid	uncompetitive	0.005 ± 0.0003 ^d^	526.3 ± 11.02 ^c^	0.009 ± 0.0004 ^b^
Ferulic acid	uncompetitive	0.008 ± 0.0004 ^b^	455 ± 9.9 ^d^	0.027 ± 0.0013 ^a^

* LOX substrate values are expressed as the mean ± SD; means with different letter superscripts (a–d) in the columns are significantly different (α = 0.05).

**Table 3:** ABTS radical scavenging ability of chosen phenolic compounds, *n =* 9.

Compound	IC_50_ [mM]
*trans*-cinnamic acid	2.12 ± 0.106 ^a^
ferulic acid	0.41 ± 0.020 ^b^
syringic acid	0.22 ± 0.011 ^c^

Values are expressed as the mean ± SD; means with different letter superscripts (a–c) in the columns are significantly different (α = 0.05).

**Table 4:** Combination index (CI) value between mixtures consisting of two polyphenols on thyroid peroxidase activity, *n* = 9.

Compound	Ferulic Acid	*trans*–Cinnamic Acid	Syringic Acid
Ferulic acid	-	0.98 ± 0.06 ^a^ Nearly additive	0.24 ± 0.05 ^c^ Strong synergism
*trans*–cinnamic acid	0.98 ± 0.06 ^a^ Nearly additive	-	0.46 ± 0.04 ^b^ Synergism
Syringic acid	0.24 ± 0.05 ^c^ Strong synergism	0.46 ± 0.04 ^b^ Synergism	-

Values are expressed as the mean ± SD; means with different letter superscripts (a–c) in the columns are significantly different (α = 0.05).

**Table 5:** Combination index (CI) value between mixtures consisting of two polyphenols on lipoxygenase LOX activity, *n* = 9.

Compound	Ferulic Acid	*trans*–Cinnamic Acid	Syringic Acid
Ferulic acid	-	0.53 ± 0.02 ^a^ Synergism	0.48 ± 0.01 ^a^ Synergism
*trans*–cinnamic acid	0.53 ± 0.02 ^a^ Synergism	-	2.15 ± 0.08 ^b^ Antagonism
Syringic acid	0.48 ± 0.01 ^a^ Synergism	2.15 ± 0.08 ^b^ Antagonism	-

Values are expressed as the mean ± SD; means with different letter superscripts (a,b) in the columns are significantly different (α = 0.05).

**Table 6:** Combination index (CI) value between mixtures consisting of two polyphenols on antiradical activity, *n* = 9.

Compound	Ferulic Acid	*trans*–Cinnamic Acid	Syringic Acid
Ferulic acid	-	0.63 ± 0.02 ^a^ Synergism	2.21 ± 0.06 ^b^ Antagonism
*trans*–cinnamic acid	0.63 ± 0.02 ^a^ Synergism	-	0.69 ± 0.03 ^a^ Synergism
Syringic acid	2.21 ± 0.06 ^b^ Antagonism	0.69 ± 0.03 ^a^ Synergism	-

Values are expressed as the mean ± SD; means with different letter superscripts (a,b) in the columns are significantly different (α = 0.05).

## Data Availability

Data reported here are available from authors upon reasonable request.
